# Circulating tumor cells in early lobular versus ductal breast cancer and their associations with prognosis

**DOI:** 10.1038/s41523-024-00623-9

**Published:** 2024-02-26

**Authors:** Silver Alkhafaji, Denise M. Wolf, Mark Jesus M. Magbanua, Laura J. van ‘t Veer, John W. Park, Laura Esserman, Rita A. Mukhtar

**Affiliations:** 1 Department of Labaratory Medicine, University of California, San Francisco, San Francisco, CA USA; 2 Department of Hematology/Oncology, University of California, San Francisco, San Francisco, CA USA; 3 Department of Surgery, University of California, San Francisco, San Francisco, CA USA

**Keywords:** Tumour biomarkers, Breast cancer

## Abstract

This is a secondary data analysis of the TIPPING study, which included 1,121 patients with stage I-III breast cancer who had enumeration of CTCs (by either CellSearch or immunomagnetic enrichment and flow cytometry [IE/FC]) and disseminated tumor cells (DTCs) at the time of surgical resection between 1999 and 2012. The primary endpoint was mean number of CTCs by histology, taking into account method of detection and treatment type, and evaluation of histology specific prognostic cutpoints. Overall, patients with ILC had significantly higher CTC counts than those with IDC, a finding which persisted in the 382 patients with CTC enumeration by IE/FC method. Additionally, among those with primary surgery, patients with ILC had significantly higher mean CTC counts than those with IDC (mean 2.11 CTCs/mL versus 0.71 CTCs/mL respectively, *p* < 0.001), which persisted on multivariate analysis. Patients with ILC and CTC-high/DTC-high status trended towards reduced DRFS HR = 9.27, 95% CI 0.95–90.5, *p* = 0.055) and had significantly decreased BCSS (HR = 10.4, 95% CI 1.07–99.7, *P* = 0.043) compared with those who were CTC-low/DTC-low. In the IDC group, CTC-high/DTC-high status was not associated with either DRFS or BCSS. In neoadjvuantly treated patients, there was no significant difference in CTC counts in the ILC group versus the IDC group (mean 0.89 CTCs/mL versus 1.06 CTCs/mL respectively, *p* = 0.82). Our findings contribute to the limited literature on CTCs and DTCs in ILC, and suggest that clinical utility and optimal thresholds for CTC and DTC assays may differ by histologic subtype in early-stage breast cancer.

## Introduction

The presence of circulating tumor cells (CTCs) in the blood of patients with breast cancer has been shown to correlate with prognosis in both the metastatic and early-stage settings^1^. As such, there is considerable interest in both identifying prognostic thresholds and understanding how dynamic changes may reflect response to therapy. Investigators have also evaluated whether tumor cells in the bone marrow or disseminated tumor cells (DTCs), provide additional prognostic information^2^. While the preponderance of data shows an association between the presence of CTCs/DTCs and breast cancer outcomes, there are mixed findings in the literature^3–13^.

We previously evaluated both CTCs and DTCs in a prospective single-institution study, the TIPPING study, in which we identified optimal thresholds for CTCs and DTCs separately and in combination in order to identify early-stage breast cancer patients with higher risk of distant recurrence or breast cancer–specific death^14^.

Recently, the presence and prognostic significance of CTCs have been shown to potentially differ by histologic subtype, with lobular histology being associated with a higher likelihood of having detectable CTCs in the early-stage setting, and being associated with higher absolute number of CTCs in the metastatic setting^15^. Indeed, a report evaluating CTCs in metastatic breast cancer patients found that prognostic thresholds differed in those with invasive lobular carcinoma compared to those with invasive ductal carcinoma^15^. These findings were hypothesized to be related to the reduced cellular adhesion in lobular breast cancer, due to the absence of the adhesion protein E-cadherin^15,16^.

Invasive lobular carcinoma (ILC) is the second most common type of breast cancer, representing 10-15% of all cases^16^. Accumulating data have identified several unique aspects of ILC, including differences in its appearance on standard imaging tools, growth pattern, response to therapy, and timing of recurrence^16,17^. While the presence of CTCs in non-metastastic breast cancer has been shown to be associated with ILC, prior analyses have not evaluated whether this is true regardless of stage^18^. Given the propensity for ILC to present at higher stages and the limited data on CTC enumeration and prognostic capability in ILC, we conducted a secondary data analysis of the prospective TIPPING cohort to compare CTC counts in ILC compared to invasive ductal carcinoma (IDC), and adjusted for stage in this non-metastatic study population. Additionally, we compared DTC levels by histologic subtype. Finally, we evaluated optimal prognostic thresholds for CTCs and DTCs, and determined whether a combined CTC/DTC count could predict worse breast cancer-specific survival in both ILC and IDC cases. Differences in CTC dynamics in ILC could have implications for the clinical application of liquid biopsies as prognostic markers and indicators of response to therapy in different histologic subtypes of breast cancer.

## Results

### CTC counts in overall population

**Fig. 1:**
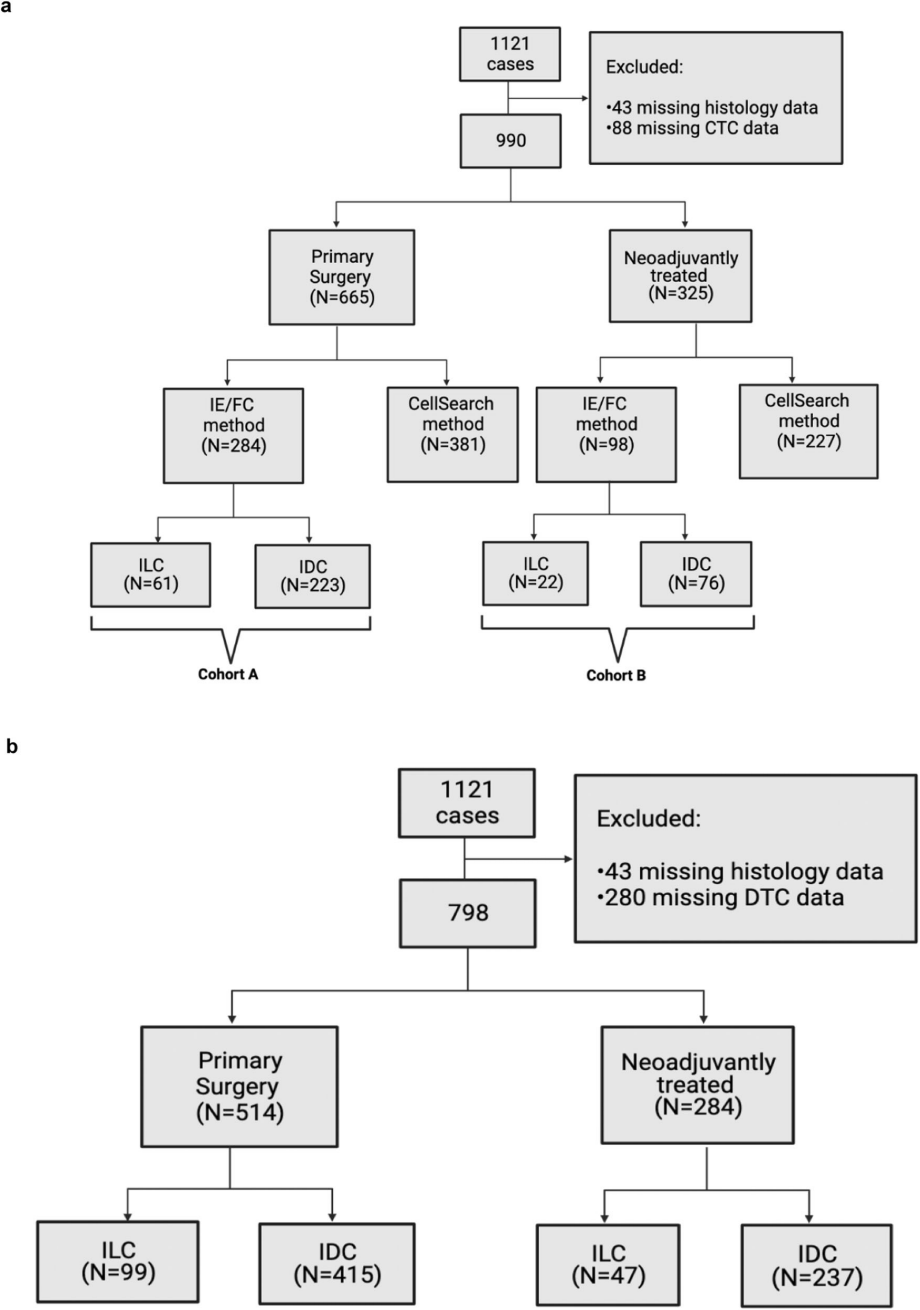
Flow diagram of patients and samples. **a** CTCs in blood were enumerated either by CellSearch (results not shown) or by IE/FC. **b** DTCs in bone marrow were enumerated by IE/FC.

Of 1,121 patients in the TIPPING study, we excluded 131 cases with missing histology or CTC counts (Fig. 1a), leaving a study population of 990 patients. The majority of patients had IDC histology (82.6%), with the remaining having ILC histology (17.4%). Overall, the mean CTC count/mL was significantly higher in those with ILC compared to IDC (0.9 versus 0.4, *p* = 0.0392). Among these cases, CTC counts were determined by the IE/FC method in 382 (38.6%) and by the CellSearch method in 608 (61.4%). The mean CTC count/mL was significantly higher in cases ascertained by the IE/FC method than CellSearch method (1.02 versus 0.09, *p* < 0.001). Additionally, patients with ILC were significantly more likely to have CTCs assessed by the IE/FC method than those with IDC (48.5% versus 36.9%, *p* = 0.006). To avoid these confounders, we evaluated CTC counts separately by enumeration method. In the 608 cases who had CTCs enumerated by CellSearch, there was no difference in mean CTC count/mL by histology (0.06 versus 0.09 in ILC and IDC respectively). Additionally, the rate of CTC positivity (>0 CTC/mL detected) did not differ by histologic subtype, with CTC positivity detected in 21.6% of ILC cases and 23.4% of IDC cases. However, in the 382 cases who had CTCs enumerated by the more sensitive IE/FC method, we identified a significantly higher mean CTC count in the ILC cohort among non-neoadjuvantly treated cases (*n* = 284), with mean of 2.11 CTCs/mL versus 0.71 CTCs/mL in ILC and IDC respectively, *p* < 0.001). We therefore further analyzed the cohort of 382 cases with CTC enumeration by IE/FC, stratified by primary surgery (cohort A, *n* = 284 patients) and neoadjuvant therapy (cohort B, *n* = 98 patients).

### Patient and tumor characteristics in cohorts A and B

In the 382 patients in cohorts A and B, 83 (22%) had ILC while 299 (78%) had IDC. Those with ILC were significantly older than those with IDC (mean age 54 versus 50, *p* = 0.003), were more likely to have hormone receptor positive, human epidermal growth factor-2 receptor negative (HR + HER2-) tumors (88% versus 66%, *p* < 0.001), had more low/intermediate grade tumors (83% versus 60%, *p* < 0.001), received neoadjvuant therapy at similar rates (27% versus 25%, *p* = 0.9), and tended to have higher stage disease (Table 1).

**Table 1:** Characteristics of the Cohort A (primary surgery) and Cohort B (neoadjuvantly treated)

Characteristic	*N*	Overall, *N* = 382^*a*^	IDC, N = 299^*a*^	ILC, N = 83^*a*^		*p*-value^*b*^
**Treatment Strategy**	382					0.9
**Cohort A (primary surgery)**		284 (74%)	223 (75%)	61 (73%)		
**Cohort B (neoadjuvantly treated)**		98 (26%)	76 (25%)	22 (27%)		
**Age (years, mean and standard deviation [SD])**	363	51 (11)	50 (11)	54 (10)		0.003
Unknown		0	0	0		
**Receptor Subtype**	368					<0.001
HR + HER2-		261 (71%)	189 (66%)	72 (88%)		
HR-/HER2-		37 (10%)	34 (12%)	3 (3.7%)		
HER2+		70 (19%)	63 (22%)	7 (8.5%)		
Unknown		14	13	1		
**Overall Pathologic Stage**	367					0.4
1		243 (66%)	196 (68%)	47 (60%)		
2		73 (20%)	56 (19%)	17 (22%)		
3		51 (14%)	37 (13%)	14 (18%)		
Unknown		15	10	5		
**Tumor Grade**	321					<0.001
1		113 (35%)	83 (34%)	30 (40%)		
2		133 (41%)	94 (38%)	39 (52%)		
3		75 (23%)	69 (28%)	6 (8.0%)		
Unknown		61	53	8		

^a^Mean (SD); *n* (%). ^b^Welch Two Sample t-test; Fisher’s exact test. Results are shown for IE/FC method.

When comparing the primary surgery group (Cohort A) to the neoadjuvantly treated group (Cohort B**)**, patients who had primary surgery were significantly older (mean age at diagnosis 53 years [standard deviation (SD) = 11], compared to 47 years [SD = 11] respectively, *p* < 0.001). Additionally, patients who had primary surgery were more likely to have earlier-stage disease, lower tumor grade, and a higher proportion of HR + HER2- subtype (76% versus 57% respectively, *p* = 0.001). In Cohort A, median follow-up times for DRFS and BCSS were 9.9, and 13.4 years, respectively; in Cohort B, median follow-up times for DRFS and BCSS were 9.2, and 12.3 years, respectively.

### Differences in clinicopathologic features by histologic type in Cohort A (primary surgery)

Of the 284 patients who underwent primary surgery, those with ILC were significantly older than those with IDC (mean age at diagnosis 56 years [SD = 10], compared to 52 years [SD = 11] respectively, *p* = 0.004). Overall stage was similar in both groups. However, ILC tumors were significantly more likely to be of lower grade than IDC tumors, and had a significantly higher proportion of the HR + HER2- subtype (92% versus 75.3% respectively, *p* < 0.001) (Table 2).

**Table 2:** Characteristics of Cohort A (primary surgery)

Characteristic	*N*	Overall, *N* = 284^*a*^	IDC, *N* = 223^*a*^	ILC, *N* = 61^*a*^	*p*-value^*b*^
**Age (years, mean and standard deviation [SD])**	284	53 (11)	52 (11)	56 (10)	0.004
Unknown		0	0	0	
**Receptor Subtype**	271				0.028
HR + HER2-		206 (76%)	153 (73%)	53 (88%)	
HR-/HER2-		21 (7.7%)	20 (9.5%)	1 (1.7%)	
HER2+		44 (16%)	38 (18%)	6 (10%)	
Unknown		13	12	1	
**Overall Pathologic Stage**	281				0.6
1		196 (70%)	157 (71%)	39 (65%)	
2		58 (21%)	44 (20%)	14 (23%)	
3		27 (9.6%)	20 (9.0%)	7 (12%)	
Unknown		3	2	1	
**Tumor Grade**	268				0.009
1		104 (39%)	77 (37%)	27 (44%)	
2		106 (40%)	77 (37%)	29 (48%)	
3		58 (22%)	53 (26%)	5 (8.2%)	
Unknown		16	16	0	

^a^Mean (SD); *n* (%). ^b^Welch Two Sample t-test; Fisher’s exact test. Results are shown for IE/FC method.

### Differences in clinicopathologic features by histologic type in Cohort B (neoadjuvantly treated)

Of the 98 neoadjuvantly treated patients, those with ILC were older than those with IDC (mean age at diagnosis 50 years [standard deviation (SD) = 11], compared to 46 years [SD = 9] respectively, *p* = 0.2). Overall stage was similar in both groups. However, ILC tumors were significantly more likely to be of lower grade than IDC tumors, and had a significantly higher proportion of the hormone receptor-positive, human epidermal growth factor-2 receptor negative (HR + HER2-) subtype (86% versus 48% respectively, *p* = 0.003) (Table 3).

**Table 3:** Characteristics of Cohort B (neoadjuvantly treated)

Characteristic	*N*	Overall, *N* = 98^*a*^	IDC, *N* = 76^*a*^	ILC, *N* = 22^*a*^	*p*-value^*b*^
**Age (years, mean and standard deviation [SD])**	98	47 (10)	46 (9)	50 (11)	0.2
Unknown		0	0	0	
**Receptor Subtype**	97				0.003
HR + HER2-		55 (57%)	36 (48%)	19 (86%)	
HR-/HER2-		16 (16%)	14 (19%)	2 (9.1%)	
HER2+		26 (27%)	25 (33%)	1 (4.5%)	
Unknown		1	1	0	
**Overall Pathologic Stage**	86				0.5
1		47 (55%)	39 (57%)	8 (44%)	
2		15 (17%)	12 (18%)	3 (17%)	
3		24 (28%)	17 (25%)	7 (39%)	
Unknown		12	8	4	
**Tumor Grade**	53				0.044
1		9 (17%)	6 (15%)	3 (21%)	
2		27 (51%)	17 (44%)	10 (71%)	
3		17 (32%)	16 (41%)	1 (7.1%)	
Unknown		45	37	8	

^a^Mean (SD); n (%). ^2^Welch Two Sample t-test; Fisher’s exact test. Results are shown for IE/FC method.

### CTC and DTC counts by IE/FC method stratified by histology

**Fig. 2:**
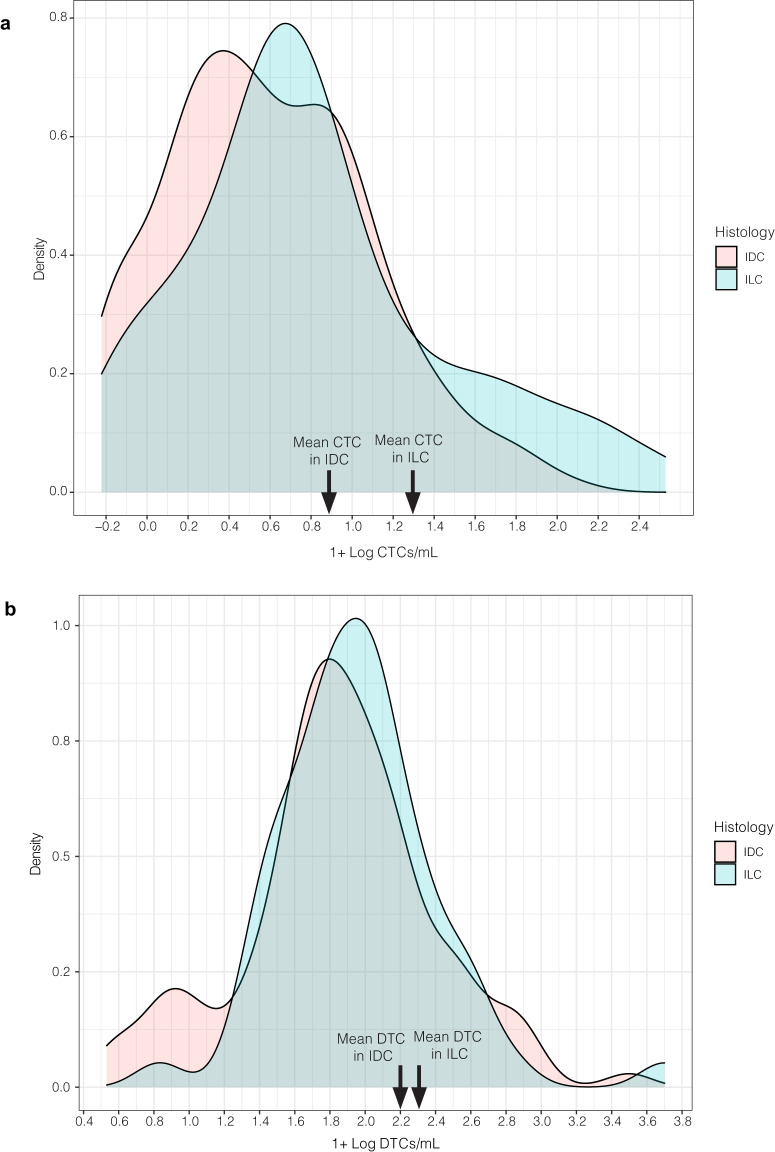
CTC counts were significantly higher in patients with ILC; there was no difference in DTC counts by histologic subtype in Cohort A (primary surgery). **a** Density plot of CTCs in ILC versus IDC histology groups. **b** Density plot of DTCs in ILC versus IDC histology groups. Blood and bone marrow samples were collected pre-operatively and were analyzed for the presence of CTCs and DTCs, respectively. For plotting purposes, “1” was added to each value and log10-transformation was performed.

In Cohort A (primary surgery), patients with ILC had significantly higher mean CTC counts than those with IDC (mean 2.11 CTCs/mL versus 0.71 CTCs/mL respectively, *p* < 0.001). This difference retained significance after adjusting for age, grade, stage, and tumor receptor subtype (*p* = 0.003) (Fig. 2a). There was no difference in the mean number of DTCs in those with ILC when compared to those with IDC (21 DTCs/mL versus 16 DTCs/mL, *p* = 0.430) (Figs. 1b and 2b). Of note, we did not find a statistical correlation between CTC counts and DTC counts in this study.

When using Monte–Carlo cross-validation to find optimal cutoffs for both CTCs and DTCs, threshold optimization yielded the following cutoffs in the ILC subset: >0.44 cells/mL for CTCs and >15.72 cells/mL for DTCs. In the IDC subset, threshold optimization yielded the following cutoffs: >0.49 cells/mL for CTCs and >8.46 cells/mL for DTCs. Among those with ILC, 50.8% (n = 31) had high CTCs (>0.44 CTCs/mL) and 21.3% (n = 13) had high DTCs (>15.72 DTCs/mL). Among those with IDC, 37.2% (*n* = 83) had high CTCs (>0.49 CTCs/mL) and 39.5% (*n* = 88) had high DTCs (>8.46 DTCs/mL).

**Fig. 3:**
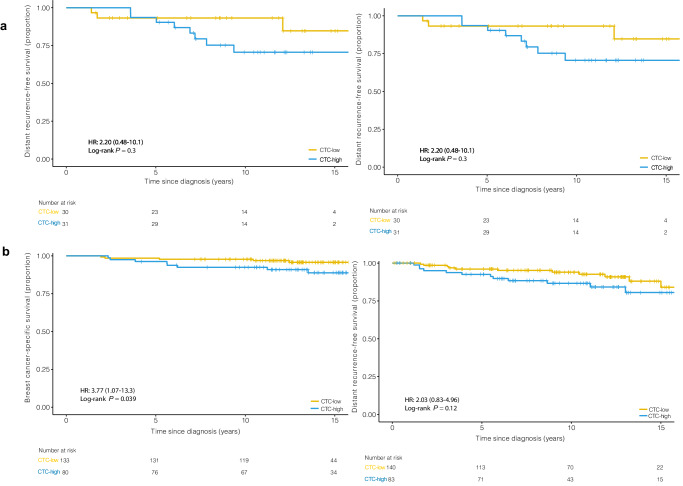
In Cohort A (primary surgery) ILC group, there was no association between CTC-high status and DRFS or BCSS. In Cohort A (primary surgery) IDC group, CTC-high status was not associated with DRFS, but CTC-high status reduced BCSS. **a** Kaplan–Meier plot for DRFS and BCSS are shown for CTCs (cutoff >0.44 CTC per mL) in ILC patients. **b** Kaplan–Meier plots for DRFS and BCSS are shown for CTCs (cutoff >0.49 CTC per mL) in IDC patients.

**Fig. 4:**
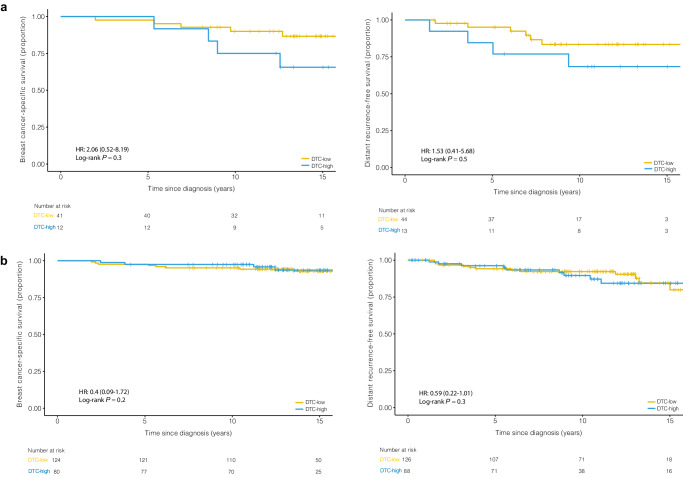
In Cohort A (primary surgery), there was no association between DTC-high status and DRFS or BCSS in either the ILC or IDC groups. **a** Kaplan–Meier plot for DRFS and BCSS are shown for DTCs (cutoff >15.72 DTC per mL) in ILC patients. **b** Kaplan–Meier plot for DRFS and BCSS are shown for DTCs (cutoff >8.46 DTC per mL) in IDC patients.

In the ILC group, there was no association between CTC-high status and DRFS or BCSS by multivariate analysis (Fig. 3a). In the IDC group, CTC-high status was not associated with DRFS by multivariate analysis. However, patients with IDC and CTC-high status had significantly reduced BCSS by multivariate analysis (HR = 3.77, 95% CI 1.07–13.3, *p* = 0.039) compared to CTC-low patients (Fig. 3b). There was no association between DTC-high status and DRFS or BCSS in either the ILC or IDC groups (Fig. 4a, b).

**Fig. 5:**
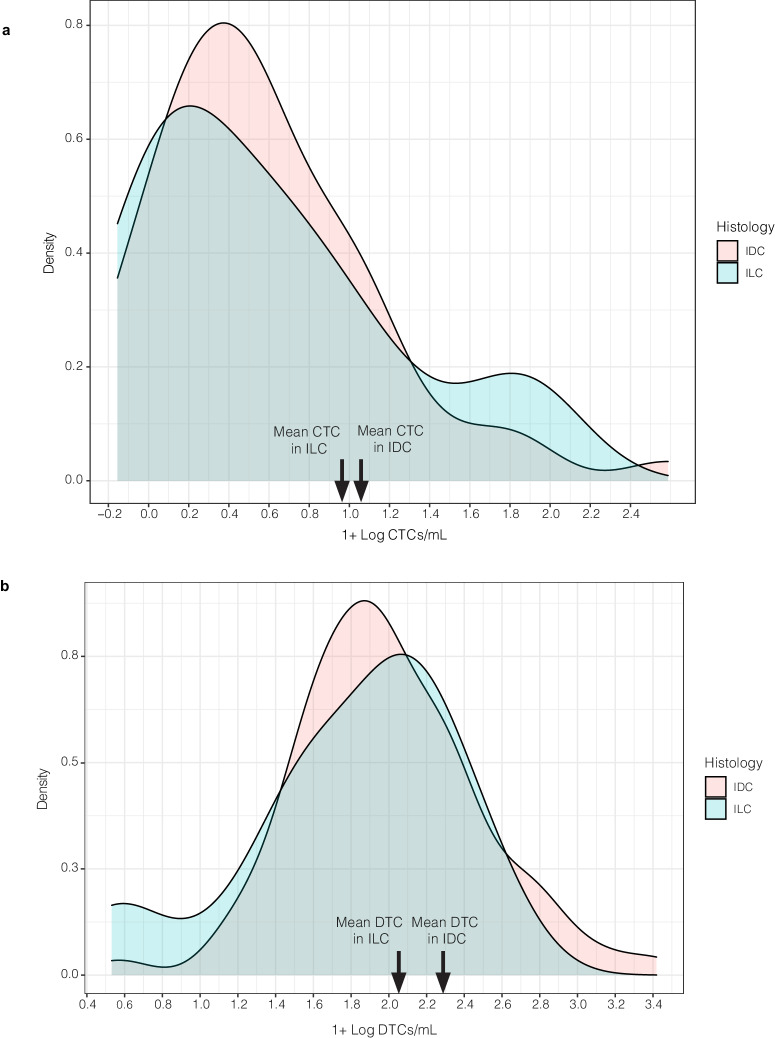
There was no significant difference in either CTC or DTC counts by histologic subtype in Cohort B (neoadjuvantly treated). **a** Density plot of CTCs in ILC versus IDC histology groups. **b** Density plot of DTCs in ILC versus IDC histology groups. Blood and bone marrow samples were collected pre-operatively and were analyzed for the presence of CTCs and DTCs, respectively. For plotting purposes, “1” was added to each value and log10-transformation was performed.

In Cohort B (neoadjuvantly treated), there was no significant diffierence in CTC counts in the ILC group versus the IDC group (mean 0.89 CTCs/mL versus 1.06 CTCs/mL respectively, *p* = 0.82). This difference was also not significant after adjusting for age, grade, stage, and tumor receptor subtype (*p* = 0.39) (Fig. 5a). There was no difference in the mean number of DTCs in those with ILC when compared to those with IDC (11.1 DTCs/mL versus 19.6 DTCs/mL, *p* = 0.085) (Fig. 5b). This difference was also not significant after adjusting for age, grade, stage, and tumor receptor subtype (*p* = 0.64).

### Combined counts of CTCs and DTCs in ILC and IDC patients in Cohort A (primary surgery)

**Fig. 6:**
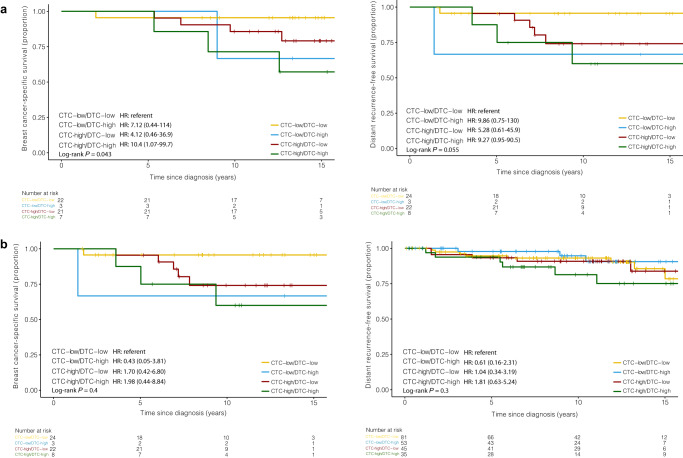
Synchronous detection of CTCs and DTCs by IE/FC identifies Cohort A (primary surgery) ILC patients with increased risk of distant recurrence and death. IDC patients have no increased risk of distant recurrence and death. **a** Kaplan–Meier plots for DRFS and BCSS are shown. In ILC patients, dichotomization into positive and negative status was based on the optimized cut-off value of >0.44 CTCs per mL and >15.72 DTCs per mL **b** Kaplan–Meier plots for DRFS and BCSS are shown. In IDC patients, dichotomization into positive and negative status was based on the optimized cut-off value of >0.49 CTCs per mL and >8.46 DTCs per mL.

In those with ILC, 13.8% of patients had high levels of both CTCs and DTCs (CTC-high/DTC-high), while 41.4% had low levels of both CTCs and DTCs (CTC-low/DTC-low). On univariate analyses, patients with ILC and CTC-high/DTC-high status trended towards reduced DRFS (HR = 9.27, 95% CI 0.95-90.5, *p* = 0.055) and had significantly decreased BCSS (HR = 10.4, 95% CI 1.07-99.7, *p* = 0.043) compared with those who were CTC-low/DTC-low. However, on multivariate analyses, CTC-high/DTC-high ILC patients had no difference in DRFS or BCSS when compared to CTC-low/DTC-low patients (Fig. 6a).

In the IDC group, 16.1% of patients had high levels of both CTCs and DTCs (CTC-high/DTC-high), while 37.3% had low levels of both CTCs and DTCs (CTC-low/DTC-low). Unlike in the ILC cohort, CTC-high/DTC-high status was not associated with either DRFS or BCSS on univariate or multivariate models in those with IDC (Fig. 6b).

## Discussion

In this secondary analysis of prospectively collected data from the TIPPING study, we found that early-stage breast cancer patients with lobular histology treated with primary surgery had significantly higher levels of CTCs compared to similar patients with ductal histology. This difference persisted when adjusting for age, overall stage, and tumor receptor subtype. We also discovered that having high levels of both CTCs and DTCs was associated with reduced BCSS in those with ILC, but not in those with IDC. In contrast, we found no difference in CTC counts by tumor histology in neoadjuvantly treated patients.

CTCs have been shown to be associated with higher tumor stage; since ILC is known to present with larger tumors and more nodal involvement, adjusting for these confounding factors is important to determine whether histology itself is associated with CTC counts^18^. In our analysis, we found that higher CTC counts in untreated ILC patients persisted even when considering the stage. The underlying cause of higher CTC counts in untreated patients with ILC is unknown, but some have hypothesized that the lack of E-cadherin in ILC results in less cohesive cells with a higher potential for hematogenous spread. However, given that one of the challenges in clinical management of patients with ILC is the decreased sensitivity of imaging studies, it is also possible that traditional methods of tumor detection result in understaging for those with ILC^19^. Such understaging could explain higher CTC counts identified in the untreated ILC cohort, but the clinical significance of this is unclear. The difficulties in accurately staging ILC with traditional imaging methods underscore the importance of conducting histologic subtype-specific analyses such as this one, as a better understanding of liquid biopsy results may ultimately assist with more accurate disease assessment for patients with diffusely growing tumors like ILC. Implementation of such findings for treatment selection and prognostication remains a clinical challenge.

Prior investigators have shown that in the metastatic setting patients with ILC have higher CTC counts than those with IDC; however, limited data exist on CTC counts and DTC counts by histology in the early-stage setting. A recent analysis showed no difference in the proportion of patients with high CTC levels by histology but did not evaluate CTC counts as a continuous value, and only included 17 ILC cases^20^. In contrast, other findings are more consistent with results in our primary surgery group; a large, pooled analysis which evaluated CTCs enumerated by the CellSearch method found that lobular histology was significantly associated with the detection of CTCs in early-stage breast cancer^18^. Interestingly, we did not find a difference in CTC counts between ILC and IDC in the subset of patients with CTCs enumerated by CellSearch. This difference may reflect a smaller sample size in our cohort; however, among the cases with IE/FC enumeration, which showed higher sensitivity in this study, CTC counts in ILC cases were significantly higher than in IDC even in a multivariable model, which has not been previously shown.

Our results in the neoadjuvantly treated cohort, where we found no difference in CTC count between ILC and IDC cases, are somewhat consistent with those of Kasimir-Bauer et al. In that analysis, patients with ILC had significantly higher DTC counts that those with IDC prior to undergoing neoadjuvant therapy; after treatment, this difference was no longer seen^5^. The finding of higher levels of either CTCs or DTCs in ILC prior to therapy in conjunction with no difference in levels after therapy may suggest eradication of circulating or disseminated cells in those with ILC, versus differences in the sensitivity of detection after therapy. Interestingly, several studies suggest decreased responsiveness to chemotherapy in ILC, which would argue against the notion of tumor eradication. Interestingly, larger studies including a meta-analysis have shown prognostic significance of CTCs in the neoadjuvant setting; the absence of this finding in our cohorts likely reflects smaller sample size^21^. These findings are hypothesis generating and should be studied further^22^.

Independent of histology, previous reports have shown conflicting results regarding the prognostic capability of CTCs or DTCs, with many showing an association between CTC/DTC positivity and survival, and other studies failing to demonstrate this^3–13^. Some investigators have shown that the detection of CTCs in patients with early-stage breast cancer is independently associated with worse distant disease-free survival, breast cancer-specific survival, and overall survival^14,18,23^. In our primary surgery cohort, we found differences in the prognostic significance of high CTCs in ILC versus IDC. Of note, prior analyses have shown a threshold of ≥ 1 CTC/mL, assessed by CellSearch method, to have prognostic significance in early breast cancer patients. In this analysis, we specifically sought to determine whether the most prognostic threshold for CTCs might differ by histology, and utilized these histology-specific cutpoints to evaluate survival outcomes in our study cohorts. Using Monte-Carlo cross-validation, we found that a threshold of 0.44 CTC/mL was most prognostic in ILC, and a similar threshold of 0.49 CTC/mL was most prognostic in IDC. The difference in this cutpoint compared to prior literature may reflect differences in CTC enumeration technique, and again smaller sample size. This likely also explains the absence of an association between DTCs and outcomes in this study, which differs from the findings of a recent large pooled analysis^7^. However, our finding of significant differences in CTC counts in ILC counts when adjusted for clinical covariates suggests that histology may need to be considered when designing assays to measure minimal residual disease, as circulating tumor cells and other liquid biopsy assays are potential markers of disease progression or treatment response in early breast cancer^24^. In our neoadjuvantly-treated group, we refrained from examining the relationship of CTCs with clinical outcomes by histologic subtype as we encountered no significant signal in CTC counts between ILC and IDC patients.

The strengths of this study include the fact that data are derived from a prospective study in which CTCs and DTCs were enumerated before surgery. A limitation, however, is that the method of enumerating CTCs changed over the course of the study. To avoid this confounding factor, we restricted our study population to the 382 patients who had CTC enumeration by the IE/FC method, stratified by treatment strategy and histology (Fig. 1). This resulted in a smaller sample size, however, still represents the largest reported cohort of ILC cases with CTC and DTC enumeration to our knowledge. Validation in an independent cohort is warranted to confirm the results of this study.

In conclusion, our findings contribute to the limited literature on CTCs and DTCs in ILC, and suggest that clinical utility and optimal thresholds for CTC and DTC assays may differ by histologic subtype in early-stage breast cancer.

## Methods

### Study population

Patients with stage I-III breast cancer who were scheduled to undergo breast cancer surgery at the University of California, San Francisco between 1999 and 2012 were recruited to participate in the TIPPING study. The TIPPING study was performed with Institutional Review Board (UCSF Committee on Human Research) approval. Informed written consent was obtained from each patient, and the study was performed in accordance with the Declaration of Helsinki.

### Study procedures

Blood and bone marrow samples were collected at the time of surgical resection from 1,121 treatment-naïve and neoadjuvantly treated study participants. Bone marrow samples were collected via a unilateral bone marrow aspiration from the posterior superior iliac crest while patients were under anesthesia prior to surgery. Two 5 mL samples were withdrawn from one site in posterior iliac crest. Peripheral blood was obtained on the same day, either in the preoperative setting or at the same time as bone marrow aspiration. Bone marrow (∼4 mL) and peripheral blood (∼10 mL) samples were drawn into tubes containing ethylenediaminetetraacetic acid for EPCAM-based method involving immunomagnetic enrichment and flow cytometry (IE/FC). Additionally, peripheral blood was collected into CellSave preservative tubes (Menarini) for CellSearch method (FDA approved in 2004), where 7.5 mL of blood was used following the manufacturer’s instructions^25^. Samples were processed within 24 hours after collection^14^.

CTCs were enumerated from blood using either the IE/FC assay (prior to August 2005) or CellSearch (starting in August 2005). DTCs were enumerated from bone marrow using the IE/FC assay. The IE/FC assay uses two distinct mAbs against EPCAM: one conjugated to immunomagnetic beads (MJ37) and the other conjugated to phycoerythrin (EBA-1) were added to whole blood or bone marrow. The sample was then placed in a magnet to capture cells labeled with the magnetic bead–antibody conjugated. The supernatant containing cells that were unbound (including red blood cells) was aspirated. Magnetic separation was repeated twice to further enrich for EPCAM-expressing cells. A nucleic acid dye (Thioflavin-T, BD Biosciences) and a mAb to the leukocyte-specific marker CD45 (2D1) conjugated to peridinin-chlorophyll-protein-Cy5.5 were added to the sample. The enriched sample was transferred to a BD TruCount (BD Biosciences) tube, and flow cytometric analysis was performed using the BD FACSCalibur (BD Biosciences). CTCs and DTCs were defined as nucleated cells that are EPCAM positive and CD45 negative.

The CellSearch method uses peripheral blood to enumerate CTCs. The samples underwent immunomagnetic enrichment using beads coated with mAb against EPCAM. Next, CTCs were detected by fluorescence microscopy. CTCs were defined as nucleated cells that are cytokeratin positive and CD45 negative. To directly compare with IE/FC, CellSearch results were expressed as CTC/mL^14^. While the range of detection has been shown to differ between the IE/FC and CellSearch methods, prior studies have shown a strong correlation between methods for CTC detection.

### Statistical analysis

Our primary aim was to determine whether patients with early-stage ILC have higher levels of detectable CTCs than those with IDC both overall and when accounting for the stage. Additionally, we compared what proportion of ILC and IDC cases had any detectable CTCs (CTC/mL > 0). We compared continuous CTC and DTC counts by histologic subtype (ILC versus IDC) in two cohorts: patients who proceeded to primary surgery, and patients who underwent neoadjuvant therapy. Additionally, we evaluated for differences in CTC level by test performed (IE/FC versus CellSearch). For these analyses, we used the Welch Two Sample t-test, Fisher’s exact test, as well as univariate and multivariate linear regression models. Univariate and multivariate Cox proportional hazards regression analyses were performed to assess the association between levels of CTCs/DTCs and DRFS and BCSS, respectively.

As a secondary aim, we identified optimal thresholds for CTC and DTC counts to predict clinical outcomes using Monte–Carlo cross-validation. This validation method was performed in R on the ILC and IDC subsets, separately. Half of the cases in each subset were used to derive a threshold between the 20^th^ and 80^th^ percentile, which returned the minimum log-rank *p-*value (maximum Kaplan–Meier curve separation) for DRFS and BCSS. This resulting threshold value was applied to the other half of the cases. Next, the log-rank *p* values were assessed in the test set; this process was repeated 1000 times. Using the logit method, the log-rank *p* values for the test set over the 1000 iterations were combined. The generated threshold with the smallest combined *p* value was then chosen. Cases with CTC or DTC counts above the optimal threshold identified by the Monte-Carlo method were classified as “CTC-high” or “DTC high,” with these cutpoints utilized to evaluate the following clinical outcomes in the non-training set cases: distant recurrence-free survival (DRFS) and breast cancer-specific survival (BCSS). To measure survival, the date of diagnosis and time of the event-of-interest were used. Survival models included covariates, such as age at diagnosis, stage, grade, and receptor subtype (Tables 1–3**)**. Patients who were lost to follow-up were censored at the time of their last visit.

Multivariate models contained the following variables: age, stage, grade, and subtype. Additionally, hazard ratios (HR) and 95% confidence intervals (CI) were calculated. Using the log-rank test, *p* values were computed. The R package “survival” was utilized for Cox proportional hazards models, Kaplan–Meier survival analysis and curves, and log-rank tests.

### Reporting summary

Further information on research design is available in the Nature Research Reporting Summary linked to this article.

### Supplementary information

Reporting Summary

## Data Availability

Aggregate data are available upon reasonable request with appropriate institutional review board approval.

## Code availability

Code for data analysis is available on https://github.com/silverjk96/ILC_IDC_CTC_DTC.git.
